# Swedish Experiences From 60 Years of Screening and Breeding Programs for Hip Dysplasia—Research, Success, and Challenges

**DOI:** 10.3389/fvets.2020.00228

**Published:** 2020-05-27

**Authors:** Åke Hedhammar

**Affiliations:** ^1^Department of Clinical Sciences, Swedish University of Agricultural Sciences, Uppsala, Sweden; ^2^The Swedish Kennel Club, Rotebro, Sweden

**Keywords:** hip dysplasia, research, screening, breeding, prevalence

## Abstract

A screening program for hip dysplasia (HD) was introduced in Sweden during the 1950s for German shepherd dogs, before for a few breeds and now any breed. Degree of canine HD was originally graded 1–4 (slight, mild, moderate, and severe) and used in Swedish screening program up to year 2000 and was thereafter replaced by letters A–E with A and B for no signs/near normal, C for mild, D for moderate, and E for severe HD. Final scoring is based on “the worst” side. In Sweden, 70% of all dogs are registered by the Swedish Kennel Club, and in relevant breeds, almost all breeding stock and 30–50% of all dogs are screened for HD. By an extensive database of all dogs registered since 1976 and mandatory identification by microchip, all results can be linked to dogs well-defined by identity and ancestral background. An implementation of structured screening and genetic health programs resulted in markedly decreased prevalence of HD already during the 1980s. The programs are based on open registries and on positive as well as negative results for identified individuals linked to their ancestral background. The successful decrease in moderate and severe HDs is illustrated for seven common breeds. However, there is also the challenge of a further decrease when already almost all breeding is performed with unaffected breeding stock. Handling that and the increased relative prevalence of less severe grades of HD (grade C) calls for breed-specific breeding strategies, taking into account the prevalence and clinical significance in each breed. Further decrease might rather be achieved by using estimated breeding values and genomic selection instead of more extensive and costly screening procedures. For the public perception of HD, the value of a clear distinction between grades D and E as a good predictor of the clinical entity vs. grade C as a tool to refine the selection criteria for breeding stock is indicated.

As one of the first countries to notice the clinical significance of hip dysplasia (HD) as a developmental disorder resulting in arthritis, active research, and actions to reduce its prevalence have now been performed in Sweden for more than 60 years.

## During the 1950s and 1960s

### Starting to Screen

Although described already in the 1930s (1), HD as a clinical entity of significance was not recognized more widely until the 1950s. Extensive research was then initiated by radiologists and geneticists from the Royal Veterinary College in Stockholm on German shepherd dogs born and raised at the breeding colony of the Swedish Armed Forces in Sollefteå (2, 3).

Although the growing puppies were repeatedly radiographically screened, primarily to predict the clinical outcome, it was soon found that a standardized screening procedure also could be used for selection of breeding stock. Since then, almost all radiological screening programs for canine HD worldwide are based on variations of that procedure. Formal screening also of privately owned dogs was organized by the Swedish Kennel Club in collaboration with the Royal Veterinary College in Stockholm in 1958 (4).

**Figure 1:**
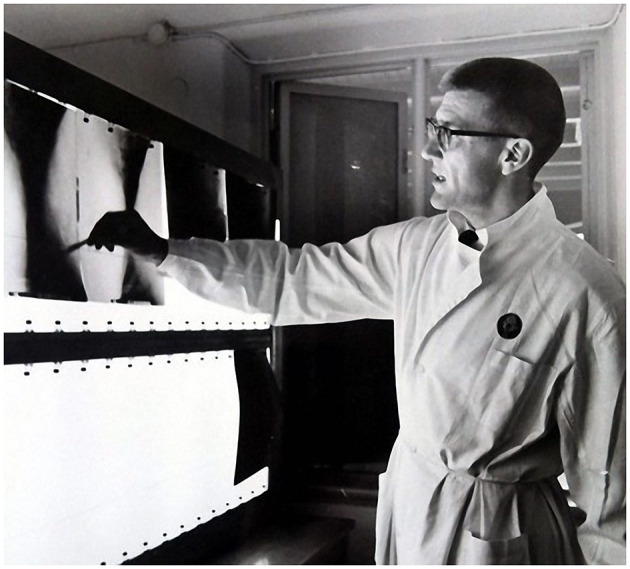
Sten-Erik Olson—a real frontier in veterinary science and diagnostic imaging with pioneering work on hip dysplasia as well as osteochondroses/elbow dysplasia.

The concept of Norberg angle as an objective measure of the fit between the femoral head and the acetabulum was introduced during the early 1960s by Prof. Sten-Erik Olson and one of his Ph.D. students—Ingmar Norberg (5). Sten-Erik Olson was a real frontier in veterinary medicine and diagnostic imaging with leading work on HD as well as on osteochondroses/elbow dysplasia (ED) (Figure 1). Ingmar Norberg was a hip panelist at that time, but then never completed his thesis and, instead, went into and is still applying his practice successfully with horses. The concept of Norberg angle has been widely applied and also criticized, but unfortunately was never formally described.

## During the 1970s

### Nutrition and Follow-Up

Based on experimental studies in Great Danes on the effect of “overnutrition” on prevalence and severity of many skeletal diseases (6), more specific studies on the detrimental effect of excessive food intake on the severity and prevalence of HD were performed in a Ph.D. project on HD by Håkan Kasström, another one of the hip panelists at that time (7).

After revealing the nutritional effects on HD, we returned (in the mid-1970s) to the Armed Forces breeding colony to explore possible changes in feeding regimes to reduce the prevalence of HD. A reduced feeding intake was instituted, but the most important finding was that despite extensive screening, the hip scoring result was not taken into account in the selection of breeding stock. An article *Hip Dysplasia and Mentality—Inheritance or Environmental* was therefore published in Sweden in 1976 (8).

In a follow-up study published in 1979 on the result of implemented breeding policies at the Armed Forces Breeding colony, we found very little selection pressure and a decrease in the prevalence of HD during the early 1960s, but there was a dramatic effect in 1973—by selecting not only the status of the Sire and the Dam but also the grandparents and littermates (9).

Base on the material in that study that included all dogs in 401 L born at the Centre from 1965 through 1973, the heritability of HD in that breeding colony was shown to be about 0.4–0.5. The results of this study were actually an early indication of the importance of estimation of breeding values—by including results also from relatives in the selection of a breeding stock for HD—which is in place almost 40 years later (10).

## During the 1980s and 1990s

### Cost Benefit

The formal implementation of a screening and health program during the late 1970s and early 1980s for HD in many breeds, and the same somewhat later for ED in some breeds, led to a significant decrease in the prevalence and severity of HD as well as ED with a positive cost benefit also in the general dog population.

Within a Ph.D. project by Lennart Swenson, a former genetic consultant to the Swedish Kennel Club, the effects of selective breeding and its economic value for the HD program operated by the Swedish Kennel Club was investigated based on 83,229 dogs from seven breeds registered by the Swedish Kennel Club born in the years 1976–1988.

A decreasing prevalence of HD, as a result of selection of breeding stock and high heritability, was found and economic analyses showed that the costs of screening and registration of coxofemoral joints were less than the value of dogs estimated to have been saved from moderate, severe, or very severe HD in six of the breeds.

It was concluded that in screening and control programs, based on an open registry with access to family records, a cost-effective decrease in the prevalence of HD can be expected and is related to the selection of the breeding stock (11). The same positive effect was also proven for elbow arthrosis (12).

Since 1986, there have now been three levels of formal genetic health programs in Sweden for all dogs to be bred in a particular breed:

1. Voluntary screening with central recording of results in open registries freely available on a public website.
2. Sire and Dam are required to have a screening result registered before breeding.
3. Sire and Dam are required to have a screening result A or B (normal hips) before breeding.

In January 2020, 137 breeds were required to have screening results for HD for both parents, out of which 38 breeds also needed both parents to be graded A or B. Results from voluntary screening were recorded in all breeds.

## During the 2000s

### Clinical Relevance

Within the scope of another Ph.D. project on HD in Sweden by Sofia Malm, a geneticist at the Swedish Kennel Club, the association between grading of hip status assessed by radiographic examination (hip screening) and the subsequent incidence of veterinary care and mortality related to HD, as well as the effects of sedation protocol on screening results, was investigated.

Screening results for hip status from the Swedish Kennel Club and data on veterinary care and mortality from the insurance company Agria were merged based on the registration number of the dog. The study populations of German shepherd, Labrador retriever, Golden retriever, Bernese mountain dog, and Rottweiler included 1,667 up to 10,663 dogs per breed insured for veterinary care and/or life in the years 1994 to 2005.

The effect of hip status at screening was highly significant (*P* < 0.001) for both life and veterinary claims related to HD in all five breeds with an increased hazard ratio (HR) for deteriorating hip status being graded 2–4 [up to year 2000 or later D–E (moderate–severe HD)] as compared with 0 and 1 [up to year 2000 or later A–C (normal hip joints–mild dysplasia)].

The conclusion was that the screening result of grades 2–4/D–E (moderate–severe HD) but not grade 1/C (mild HD) is a good predictor of clinical problems and that selection based on the screening results for hip status can be expected to reduce the risk of HD-related clinical problems (13).

### Sedation

To investigate the effect of sedation method on the screening results for HD and ED, a questionnaire survey of routines for hip and elbow screening at Swedish veterinary clinics was related to the results of hip and elbow status for eight breeds (Bernese mountain dog, Boxer, German shepherd dog, Golden retriever, Labrador retriever, Newfoundland, Rottweiler, and St Bernard). A total of 5,877 and 5,406 dogs with a screening result for HD and ED, respectively, were included. The type of chemical restraint used for sedation was shown to have a strong effect on the screening result for HD but not for ED (14). Neuroleptics such as acepromazine was shown to reveal fewer signs of HD than products resulting in heavier sedation.

Following the results of this study, recording of the type of chemical restraint used for sedation during hip screening became mandatory in Sweden. This also made it possible to account for the effect of the sedation method in a model for the estimation of breeding values, EBVs, for HD.

Since 2020, the sedation method when screening for HD has been regulated to not be performed with just neuroleptics such as acepromazine.

### Further Studies

During the 2000s, additional Swedish studies have further revealed the effects of diet, weight, and body condition scores (BCSs) as risk factors for HD (15–17).

In ongoing studies, the effects of weight and BCS on health including HD are being further explored (17).

### Estimated Breeding Values

Further decreasing the prevalence of HD in populations that are already on mandatory phenotypic screening and even mostly free from any signs of HD calls for more refined selection tools. Estimated breeding values (EBVs) for many breeds have therefore gradually been introduced in Sweden since 2012 (13). Each dog's EBV is calculated by linking pedigree information with data from the registrations of hip status, allowing the genetic risk to be calculated for every individual in the pedigree. EBVs are computerized and updated every week currently (2020-01-01; https://hundar.skk.se/avelsdata/Initial.aspx) for 44 breeds. Also the possibility of combining data for international genetic evaluation has been outlined (10, 18, 19).

### International Efforts

Since the 1960s, Nordic hip panelists gather twice yearly to calibrate the procedure and evaluation criteria and have been actively involved in further international standardizations at meetings organized by Fédération Cynologique Internationale (FCI) in 1981 in Dortmund and later in 2007 in Copenhagen.

During the 1990s, great efforts were undertaken by the World Small Animal Veterinary Association (WSAVA) and FCI to harmonize the programs by FCI, Orthopaedic Foundation (OFA), and the British Veterinary Association (BVA)/Kennel Club (KC) (20).

### International Hip Panel Meeting in Copenhagen 2021

To harmonize and validate the evaluation and scoring of radiographs, a meeting for actively operating HD panelists will be arranged by the FCI in Copenhagen on September 9–10, 2020. As in former meetings of that kind-−1981 in Dortmund and 2007 in Copenhagen—Swedish and other Nordic panelists will take an active part in planning, running, and following up on it.

### Evaluation

In 2019, an extensive evaluation of the Swedish Hip Dysplasia Program was initiated by the board of the Swedish Kennel Club and performed by internal and external reviewers.

The evaluation showed an early successful decrease in all grades of HD followed by a later much slower decrease in affected HD phenotype despite an improved genetic trend.

These findings could be explained by the initial change from usage of unscreened and affected to almost exclusively screened and unaffected breeding stock, and later less selection pressure from phenotypic selection due to less variation (i.e., a larger proportion of dogs scored as normal).

A relative increase in grade C (mild dysplasia) in the later period was partly explained by the shift from the use of less to more effective sedation and by the increased use of digitally submitted radiographs.

Based on the findings in the evaluation of sedation, acepromazine as the only preparation is no longer allowed, and only digitally submitted radiographs will be accepted.

### Molecular Genetics

The availability of extensive recordings of hip status in Swedish dogs and the use of genome-wide association studies already reported (21, 22) as well as ongoing molecular genetic studies on various phenotypes in the same population have formed the basis also for molecular genetic studies on HD. Recently obtained funding will make it possible to investigate the molecular genetic features of HD in Swedish dogs well-defined by identity and ancestral background.

## Further Perspectives

Having initiated the extensive screening and health programs for HD based on a simple phenotypic screening of individual dogs, it is now a Swedish responsibility to further develop methods to maintain and enable a further decrease in the prevalence of HD. That might rather be achieved by usage of EBVs and possibly also genomic selection instead of more extensive and costly screening procedures. Selection for HD and other health problems is, however, hampered by the fact that these are rarely the prime selection criteria in pedigree dogs.

## Success

**Figure 2:**
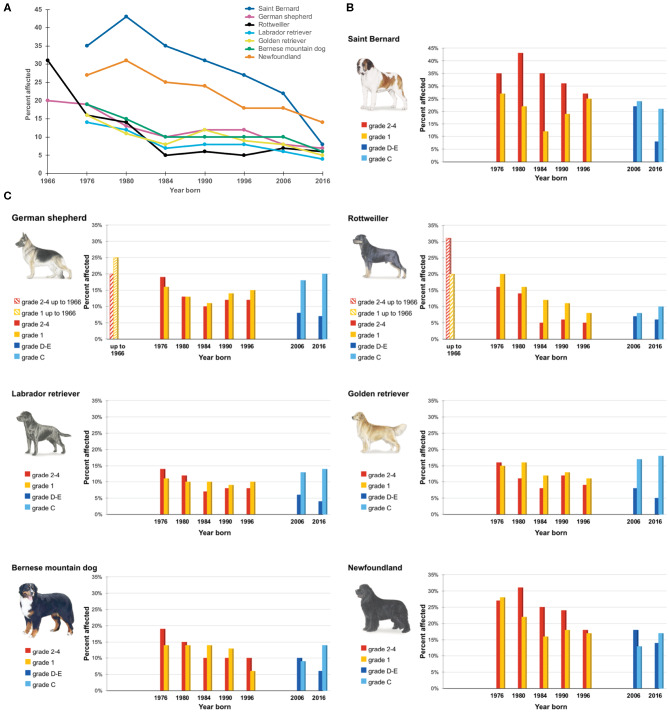
Degree of canine hip dysplasia is graded 1–4 (slight, mild, moderate, and severe) up to year 2000 and thereafter by letters A–E, with **(A,B)** for no signs/near normal, C for mild, D for moderate, and E for severe hip dysplasia. Final scoring is based on “the worst” side. Prevalence of Hip Dysplasia for seven breeds in selected birth cohorts 1976–2016 (since the 1960s for German shepherd and Rottweilers). **(A)** The decline in the percentage of dogs diagnosed with grades 2–4/D–E of HD over the last 50 years for seven breeds. **(B)** Percentage of Saint Bernard dogs diagnosed with different grades of HD over the past 50 years. **(C)** Percentage of dogs from six additional breeds diagnosed with different grades of HD over 50 years (please note the difference in the y-axis scale compared to **(B)**.

The screening programs introduced in Sweden already during the late 1950s made it possible to select screened and unaffected dogs for breeding. That possibility was rewarded and even requested by applied breeding programs from 1984. By using the genetic health programs instituted during the 1980s, a dramatic decrease in the number and fractions of dogs graded as moderate and severe could be achieved. The successful reduction of HD in Swedish dogs since more than 60 years is well-illustrated in Figure 2.

By applied selection, the prevalence of moderate to severe HD has been reduced to one third in all of the most commonly predisposed breeds. The data in Figure 2 are composed of published data on dogs born in the years 1976–1984 (11) and data freely available online for all breeds through the SKK database (https://hundar.skk.se/avelsdata/Initial.aspx). For German shepherd dogs and Rottweilers, data also for the very first dogs screened up to 1966 are available.

Today in Sweden the prevalence of moderate and severe HD (D and E) in most breeds is lower than 10% (German shepherd dogs 7%, Labrador retrievers 4%, Golden retrievers 5%, Bernese mountain dogs 6%, Rottweilers 6%). It is only in a few giant breeds with a few registered dogs and no restrictions on breeding stock that the prevalence of grades D and E is over 10%.

The successful reduction of HD is based on extensive screening mandatory regulated by the Swedish Kennel Club with the results in an open registry including positive as well as negative results and the wide use of this information in the selection of the breeding stock. Since the 1980s, majority of the breeding in most dog breeds are done with both parents screened and free of HD (Grade 0/A or B).

The strength of the results is that the data represent the majority of the dogs of affected breeds in Sweden and that such a high proportion of these are screened for HD. In most predisposed breeds, not only breeding stock but also the majority of other dogs within the breed are screened, adding valuable information for the estimation of breeding values.

The achieved results are to be compared with other breed populations even in countries with long-lasting, but not as extensive, screening programs as in Sweden, e.g., the US, Switzerland, and UK (22–25).

## Challenges

In many breeds with generations of breeding stock with normal hip status since the 1980s, it is challenging to achieve a further decrease at the same rate using phenotypic selection.

To achieve further decrease in the number and fractions of severe HD, breed-specific strategies are needed based on the structured usage of estimated breeding values.

A somewhat increased prevalence of dogs with mild HD (grade C) in later years—likely caused by usage of sedation restraints and digital radiology revealing more—has to be taken into account in breed-specific breeding strategies. This is partly accounted for in the prediction of EBVs. In a holistic approach, breed-specific prevalence of HD should be balanced with other health problems within each breed.

There is a value in it and a challenge to influence the public perception of the various grades of HD at screening. A clear distinction between grades D and E as a good predictor of the clinical entity vs. grade C as a tool to refine the selection criteria for breeding stock would reduce the stigma of individual dogs graded C being “diagnosed” as hip dysplastic. It would still stress the value of indicating dogs graded C in the selection of breeding stock.

## Author Contributions

The author contributed to the conception or design of the work, drafting and revising, and final approval of the version to be published.

## Conflict of Interest

The author declares that the research was conducted in the absence of any commercial or financial relationships that could be construed as a potential conflict of interest.
